# A Fast Neural Network Approach to Predict Lung Tumor Motion during Respiration for Radiation Therapy Applications

**DOI:** 10.1155/2015/489679

**Published:** 2015-03-29

**Authors:** Ivo Bukovsky, Noriyasu Homma, Kei Ichiji, Matous Cejnek, Matous Slama, Peter M. Benes, Jiri Bila

**Affiliations:** ^1^Department of Instrumentation and Control Engineering, Faculty of Mechanical Engineering, Czech Technical University in Prague, 16607 Prague, Czech Republic; ^2^Department of Radiological Imaging and Informatics, Graduate School of Medicine, Tohoku University, Sendai 980-8575, Japan; ^3^Division on Advanced Information Technology, Yoshizawa Laboratory, Tohoku University, Sendai 980-8578, Japan

## Abstract

During radiotherapy treatment for thoracic and abdomen cancers, for example, lung cancers, respiratory motion moves the target tumor and thus badly affects the accuracy of radiation dose delivery into the target. A real-time image-guided technique can be used to monitor such lung tumor motion for accurate dose delivery, but the system latency up to several hundred milliseconds for repositioning the radiation beam also affects the accuracy. In order to compensate the latency, neural network prediction technique with real-time retraining can be used. We have investigated real-time prediction of 3D time series of lung tumor motion on a classical linear model, perceptron model, and on a class of higher-order neural network model that has more attractive attributes regarding its optimization convergence and computational efficiency. The implemented static feed-forward neural architectures are compared when using gradient descent adaptation and primarily the Levenberg-Marquardt batch algorithm as the ones of the most common and most comprehensible learning algorithms. The proposed technique resulted in fast real-time retraining, so the total computational time on a PC platform was equal to or even less than the real treatment time. For one-second prediction horizon, the proposed techniques achieved accuracy less than one millimeter of 3D mean absolute error in one hundred seconds of total treatment time.

## 1. Introduction

In radiation therapy, accurate and sufficient amount of dose delivery only to the target tumor is required to not only maximize the therapeutic effects, but also minimize inaccurate delivery of doses to healthy tissues surrounding the tumor. Such accurate irradiation is, however, a nontrivial task due to the body motion. For example, the respiratory motion complicates the targeting of external radiation to tumors in lungs, pancreas, and other thoracic and abdominal sites. The tumor motion can be associated with the internal movements caused by respiration and cardiac cycles and also with systematic drifts and patient's stochastic movements [1, 2]. Among them, respiration is dominant and thus the respiratory motion has been widely analyzed. In lung tumor motion, it is well known to have amplitude between 0.5 and 2.5 cm, even some times 5 cm [3]. As a consequence, the dose distribution may be delivered significantly different from the prescribed one and increase the radiation toxicity dramatically [4–9]. The time series of the lung respiration has a quasiperiodic nature and the behavior may vary in time [2, 5, 10]. The respiration motion becomes a complex nonstationary process; that is, it changes amplitude and period over time. Some breathing is highly irregular in patients whose pulmonary functions are affected by disease [11–14]. Several methods have been developed for the respiratory motion gated radiation therapy or real-time tumor tracking, but their use is still questioned [2, 10]. Three general approaches have been achieved to predict respiration behavior [10].

In Isaksson et al. [5] it is shown that adaptive signal processing filters can provide more accurate tumor position estimates than stationary filters when presented with nonstationary breathing motion. Murphy and Dieterich [10] analyzed linear versus nonlinear neural network filter for predicting tumor motion when breathing behavior is moderately to extremely irregular. In Homma et al. [15] the authors developed a time series prediction based on a seasonal ARIMA model by using the real-time compensation of the time variant nature involved in the cyclic dynamics of the respiration motion. Their evaluation by using a clinical dataset showed that the proposed system can achieve a clinically useful high accuracy and long-term prediction of the average error 1.05 ± 0.99 mm at 1-second prediction ahead. Riaz et al. [16] proposed a linear adaptive filter with gradient descent and support vector regression approach to predict tumor movement up to 1 sec into the future. They used data from 14 treatment sessions and a root mean square error (RMSE) was used as a metric. Their support vector regression gave them the best prediction accuracy for 400 ms and 1 sec, with RMSE less than 2 mm at 1 s. In Ichiji et al. [17] the authors proposed a tumor motion prediction using time variant seasonal ARIMA (TVSARIMA) model; they took attention in estimating the time variant periodical nature of lung tumor motion. In order to obtain better prediction accuracy, Ichiji et al. [17] combined TVSARIMA with three more methods: unweighted average, multiple regression, and multilayer perceptrons (MLPs) type of neural network (NN). The authors reached the highest prediction accuracy by using combination of TVSARIMA and MLP with 10 neurons in a hidden layer and the mean absolute error was 0.7953 ± 0.0243 mm at 0.5 s ahead and 0.8581 ± 0.0510 mm for 1-second prediction horizon. Yan et al. [7] presents an adaptive approach to infer internal target position by external marker positions. For both internal and external marker motions, two networks with the same type were used. During a simulation, a patient was immobilized and positioned as if it were in a treatment room. The authors indicated that their technique was capable of predicting target position for short-term response time (less than 10 ms). They achieved prediction error 23% on average of internal target positions based on the clinical data observed between external marker and internal marker motions. In Ma et al. [3] a tumor position was detected by an electronic portal imaging device. The methods used are adaptive filtering and nonlinear method based on Takens theorem. The adaptive filtering algorithm is fast whilst the strategy based on nonlinear time series analysis approaches better precision with the price of higher computational effort. In Murphy [18], neural networks are analyzed to correlate surrogate and tumor motion temporal prediction to compensate the lag time of tracking system; when the correlation changes rapidly with time, the simple stationary linear filter was unable to make a useful prediction, while the neural network provided the best prediction of the data with time changing correlation.

From the above reviewed achievements, it is apparent that feedforward NNs or MLPs have promising capabilities for implementation to lung motion time series prediction, and lung motion prediction with NN is a subject of great interest in medicine due to the possibility of capturing dynamics and structural aspects [4, 10]. Some authors are convinced that deep analysis is still needed [4, 10, 16, 19].

From the theoretical point of view, we shall also recall the publication of Hornik et al., 1989 [20], where it is presented that MLP can approximate a function to an arbitrary degree of accuracy that has become often cited in publications on NNs by many authors up to nowadays; however, it is not usually mentioned explicitly that the statement about arbitrary degree of accuracy of MLPs is limited only to training data because the very precise training does not necessarily imply correct functionality of the trained NN for new data, that is, for testing. Then we talk about the well known issues such as generalization capability, overfitting (overtraining) issue, or about the local minima issue of MLPs that makes proper training of NNs, especially for nonstationary data such as lung motion, a nontrivial issue.

Regarding the above mentioned issues of MLPs and considering our experience with higher-order nonlinear neural architectures we also extend our study with focus on a second-order nonlinear neural unit which is the so called quadratic neural unit (QNU) [21–25]. QNU can be considered a standalone second-order neural unit of higher-order NNs (HONN) or a class of polynomial NNs [26–28]. For fundamental works on higher-order NNs we can refer to works of [29–34]. We may recall that polynomial neural networks (including QNU) are attractive due to the reliable theoretical results for their universal approximation abilities according to the Weierstrass theorem and for their generalization power measured by the Vapnik-Chervonenkis (VC) dimension [27].

For the fact we study implementation of static NNs, we use the most popular learning algorithm; that is, the Levenberg-Marquardt (L-M) algorithm [35, 36] that is a powerful optimization algorithm and it is easy to be implemented. L-M technique is used for nonlinear least-squares problems. We also briefly compare the performance of a classical gradient descent (GD) adaptation algorithm with the best performing predictor in our experiments. Also, because of the nonstationary nature of lung tumor motion in time, we implemented sliding window retraining (e.g., [37, 38]) to capture temporal variations in time series validity of the neural model at every sample of prediction.

In this paper, we propose and study prediction method of lung tumor motion, first, with the use of conventional static MLP with a single hidden perceptron layer and, second, with the static QNU, that is, a class of polynomial neural network (or a higher-order neural unit). We also demonstrate that QNU can be trained in a very efficient and fast way for real-time retraining. The objective of our study was to achieve the prediction accuracy within 1 mm for prediction horizon *t*_pred_ = 1 second by using NN approaches and to study capabilities of the simplest yet powerful NN models. That is, we adopt static MLPs and QNUs to achieve better prediction accuracy than in published and comparable works that are referenced above. The QNU was chosen for its high quality of nonlinear approximation and its excellent convergence due to its in-parameter linearity that implies a linear optimization problem while the predictor is nonlinear [23].

Section 2 describes 3D lung tumor motion data used for the experimental study.  Section 3 describes the NN models, that is, the MLP and QNU, the real-time retraining technique, the used L-M and GD learning algorithms, and the modifications of the L-M learning algorithm (later as MLM) to increase efficiency and the speed of the retraining for real-time computation.  Section 4 presents results with real lung tumor 3D motion data, and these are discussed in Section 5 with directions for further research on unexpected move processing, on increasing the accuracy via online estimation accuracy with connotations to intensity modulated approach. At the very end, more results are shown also on additional artificial time series featuring respiration nonlinear dynamics and unexpected move in Appendix, where also the evidence of lower accuracy of linear predictor is shown for both artificial and real data.

## 2. Data Description

**Figure 1:**
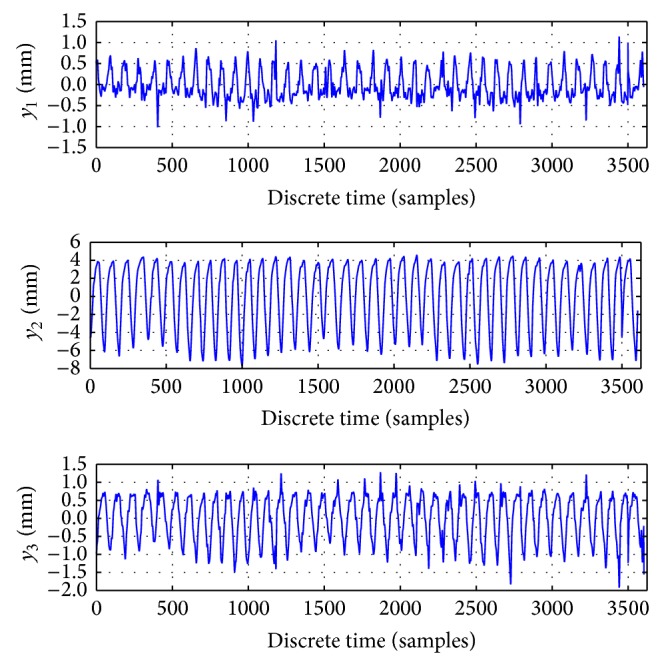
Preprocessed time series of the observed lung tumor marker position. The sampling frequency *f* = 30 Hz.

The three-dimensional time series of lung tumor motion data (Figure 1) with uncontrolled respiration of a patient were obtained courtesy of Hokkaido University Hospital. To measure the three-dimensional coordinates of the tumor motion as shown in Figure 1, a fiducial gold marker was implanted into the lung tumor or its neighbour, and the motion was measured in the directions of the lateral, cephalocaudal, and anteroposterior axes, respectively [15, 17]. The original sampling frequency was 30 Hz, and the spatial resolution was 0.01 mm; the time series were preprocessed by applying Kalman filter and statistical filters in order to reduce the noise and avoid abnormal data included in rough data of the time series [15, 17, 39].

The elements of vector **y**(*k*) are

(1) $$\begin{array}{c} y\left(k\right)=\left[\begin{array}{ccc} y_{1}\left(k\right) & y_{2}\left(k\right) & y_{3}\left(k\right) \end{array}\right]. \end{array}$$

The dominant periods of the time series are varying around 3 seconds.

## 3. Prediction Methods

This section describes the neural network models used in this study.  Section 3.1 gives the necessary details on the sliding window retraining technique that increased prediction accuracy (as discussed in Section 5 later).  Section 3.2 gives details on the implemented classical perceptron-type static feedforward NN with a single hidden layer of neurons with sigmoidal output function (Figure 3) and the L-M algorithm used for batch training of this neural architecture is recalled.  Section 3.3 presents weight-by-weight modification of L-M that accelerates real-time computation by avoiding inverse matrix computation.  Section 3.4 describes the implemented static QNU (Figure 4, equations (11) and (13)) that performs nonlinear input-output mapping yet its linear optimization nature suppresses the issue of local minima for convergence of neural weights. Also modification of the L-M algorithm (9)–(14) for enhanced computational speed of QNU is described as the inverse matrix computation is avoided and the Jacobian is constant for static QNU.

### 3.1. Sliding Window Retraining

**Figure 2:**
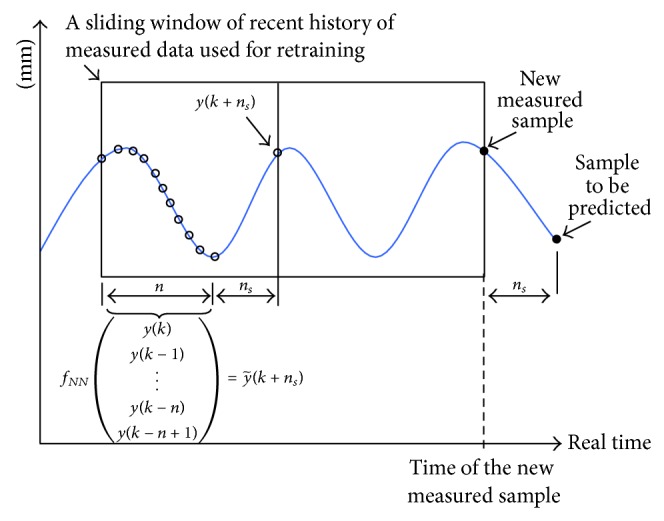
The principle of sliding (retraining) window for model retraining at every new measured sample, the window slides ahead with each new measured sample. The total length of a (re)training window is denoted by *N*_train_.

**Figure 3:**
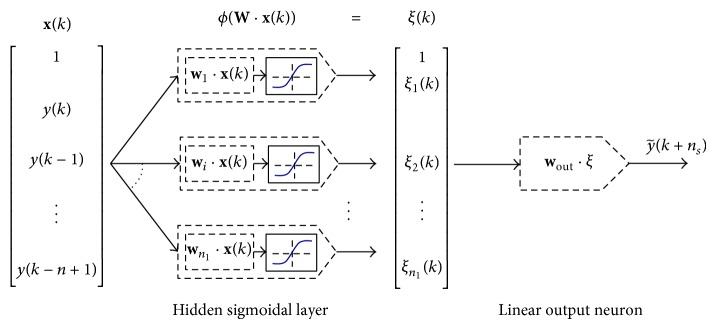
The used static feedforward perceptron-type NN as implemented for time series (direct) prediction.

**Figure 4:**
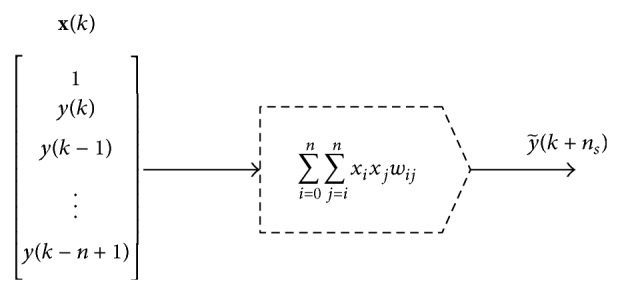
Static QNU architecture with *n* external inputs (real measured values) as implemented for time series (direct) prediction.

Because the respiration time series are naturally nonstationary and thus quasiperiodic with time varying frequency, mean, and amplitudes, it is impossible to obtain a generally valid model from a single training data set. Therefore, we investigated the effect of real-time retraining of the above described predictive models (Figures 3 and 4) to their prediction accuracy. By retraining with the most recent history of measured values, we capture the contemporary valid governing laws of a nonstationary system. We retrained the models at every new measured sample, that is, before each new sample prediction. This approach can be referred to as a sliding window approach (e.g., [37, 40]). Before NN retrainings, every sliding window was normalized by subtracting the mean and divided by standard deviation, respectively, for each signal (**y**_1_, **y**_2_, **y**_3_). The retraining (sliding) window for the predictive models (Figures 3 and 4) is shown in Figure 2, where *f*_NN_ stands for the mapping function of the NN model (MLP or QNU).

After the current window training is performed, the NN predicts the unknown *n*_*s*_ samples ahead from the new measured value and then the data normalization, retraining, and prediction repeat when a new sample is available.

### 3.2. Perceptron Neural Network with Levenberg-Marquardt Adaptation

The static MLP NN with discrete time notation *k* and with a single hidden layer is given by the following equation:

(2) $$\begin{array}{c} \tilde{y}\left(k+n_{s}\right)=w_{\text{out}}\cdot \xi \left(k\right)=w_{\text{out}}\cdot \phi \left(W\left(k\right)\cdot x\right), \end{array}$$

where $\tilde{y}(k+n_{s})$ is the output of the network calculated at time *k* as an *n*_*s*_ samples ahead predicted value. **W** is a weight matrix whose rows correspond to weights of neurons in a hidden layer, *w*_out_ is a weight vector for the output neuron, and the input vector **x** is given for the static model (2), that is, for a directly predicting (static) model as

(3) $$\begin{array}{c} x\left(k\right)=\left[\begin{array}{c} 1\\ y\left(k\right)\\ y\left(k-1\right)\\ \vdots \\ y\left(k-n+1\right) \end{array}\right], \end{array}$$

where the length of input vector **x** is *n* + 1, and the sigmoidal output function of neurons in the hidden layer is given as follows:

(4) $$\begin{array}{c} \phi \left(\nu \right)=\frac{2}{1+\exp\left(-\nu \right)}-1. \end{array}$$

This network architecture (2)–(4) with *n*_1_ neurons in its hidden layer is sketched in Figure 3, and this model was studied as a classical NN model for direct prediction of time series in Figure 1 with sliding window retraining was described in Section 3.1.

The common formula for L-M algorithm for weight increments of the *i*th hidden neuron at every epoch of training is then given as follows:

(5) $$\begin{array}{c} \Delta w_{i}={\left[J_{i}^{T}\times J_{i}+\frac{1}{\mu }\times I\right]}^{-1}\times J_{i}^{T}\times e, \end{array}$$

where elements of Δ**w**_*i*_ are weight increments of the *i*th neuron, **I** is (*n* + 1)×(*n* + 1) identity matrix, *μ* is a learning rate that is optionally adjustable (see below), **e** is a vector of errors between real values and neural outputs (7), *T* stands for matrix transposition, and **J**_*i*_ is the Jacobian matrix that contains the first derivatives of the network outputs with respect to weights of the *i*th neuron as follows:

(6) $$\begin{array}{c} J_{i}=\left[\begin{array}{cccc} \frac{\partial \tilde{y}\left(k=1\right)}{\partial w_{i,0}} & \frac{\partial \tilde{y}\left(1\right)}{\partial w_{i,1}} & \cdots & \frac{\partial \tilde{y}\left(1\right)}{\partial w_{i,n}}\\ \frac{\partial \tilde{y}\left(2\right)}{\partial w_{i,0}} & \frac{\partial \tilde{y}\left(2\right)}{\partial w_{i,1}} & \cdots & \frac{\partial \tilde{y}\left(2\right)}{\partial w_{i,n}}\\ \vdots & \vdots & \ddots & \vdots \\ \frac{\partial \tilde{y}\left(N\right)}{\partial w_{i,0}} & \frac{\partial \tilde{y}\left(N\right)}{\partial w_{i,1}} & \cdots & \frac{\partial \tilde{y}\left(N\right)}{\partial w_{i,n}} \end{array}\right], \end{array}$$

where *N* is the length of training data (the number of samples). A training performance is for *N* training samples given as the sum of square errors

(7) $$\begin{array}{c} Q\left(\text{epoch}\right)=\overset{N}{\underset{k=1}{\sum }}e{\left(k\right)}^{2},\;\text{where}\;\;e\left(k\right)=y\left(k\right)-\tilde{y}\left(k\right). \end{array}$$

The L-M algorithm for the perceptron-type network as in (2)–(4) (Figure 3) requires computation of the Jacobian matrix **J**_*i*_ as in (6) at each epoch, so the matrix inverse has to be always calculated according to the basic L-M formula (5) epoch times. The inverse matrix calculation as in (5) for the network (3) results in slowing down the real-time computation. Modified Levenberq-marquard algorithm is able to avoid that and the retraining and prediction run faster; this is presented in Section 3.3.

### 3.3. Perceptron Neural Network with Modified Levenberg-Marquardt Adaptation

The resulting formula for modified L-M algorithm for the *j*th weight increment of the *i*th hidden neuron at every epoch of training is then given as follows:

(8) $$\begin{array}{c} \Delta w_{i,j}={\left[j_{i,j}^{T}\times j_{i,j}+\frac{1}{\mu }\times I\right]}^{-1}\times j_{i,j}^{T}\times e, \end{array}$$

(9) $$\begin{array}{c} \Delta w_{i,j}=\frac{j_{i,j}^{T}}{j_{i,j}^{T}\times j_{i,j}+1/\mu }\times e={\tilde{j}}_{i,j}\times e, \end{array}$$

where Δ*w*_*i*,*j*_ is a *j*th weight increment of the *i*th neuron, **I** is (*n* + 1)×(*n* + 1) identity matrix, *μ* is a learning rate,** e** is a vector of errors between real values and neural outputs (7), *T* stands for matrix transposition, and **j**_*i*,*j*_ is the Jacobian vector:

(10) $$\begin{array}{c} j_{i,j}=\left[\begin{array}{c} \frac{\partial \tilde{y}\left(1\right)}{\partial w_{i,j}}\\ \frac{\partial \tilde{y}\left(2\right)}{\partial w_{i,j}}\\ \vdots \\ \frac{\partial \tilde{y}\left(N\right)}{\partial w_{i,j}} \end{array}\right] \end{array}$$

that contains the first derivatives of the network outputs with respect to *j*th weight of the *i*th neuron as follows. Perceptron neural network predictor with this modification of Levenberg-Marquardt learning algorithm is further denoted as MLP predictor with MLM learning.

In next subsection we show QNU and its linear nature of optimization (by L-M algorithm) that, in principle, prevents QNU from local minima issue for a given training data set, so the weight convergence of QNU is superior to conventional perceptron-type neural networks [23, 41].

### 3.4. Quadratic Neural Unit with Levenberg-Marquardt Adaptation

QNU may be considered as a special case of higher-order neural unit or as a case of polynomial NN. The static QNU is sketched in Figure 4.

The output of QNU from Figure 4 can be written in a vector multiplication form that can be decomposed into a long vector representation as follows:

(11) y~k+ns=∑i=0n∑ j=0nxixjwi,j=w0,0x02+w0,1x0x1+⋯+wi,jxixj+⋯+wn,nxnxn=w·colx,

where *x*_0_ = 1 (as shown in Figure 4), $\tilde{y}$ is a predicted value, *x*_1_, *x*_2_,…, *x*_*n*_ are external neural inputs at a sample time *k*, *w*_*i*,*j*_ are neural weights of QNU, **w** is a long-vector representation of weight matrix of QNU, and** colx** is a long column vector of polynomial terms of neural inputs defined as follows:

(12) colxk=xikxjk:i=0…n, j=i…n,where  x0=1.

Notice that for weight optimization of polynomial static model (11), all *x*_*i*_ and *y*(*k* + *n*_*s*_) are substituted with measured training data, so (11) yields a linear combination of neural weights that has, in principle, a unique solution for a given training data. Thus, contrary to MLP networks, the linear optimization nature of QNU implies that QNU avoids the local minima issue for a given training data while the neural model maintains high quality of nonlinear approximation that we have observed so far [23, 41].

Another advantage of QNU against MLP is the fact that the Jacobian matrix of QNU, that is, **J**, derived accordingly to (6), becomes merely function of inputs; thus the **J** of QNU becomes a constant for its all training epochs. Then, **J** of QNU is given for its all weights as follows:

(13) $$\begin{array}{c} J=\left[\begin{array}{cccccc} 1 & x_{1}\left(k=1\right) & \cdots & x_{i}\left(1\right)x_{j}\left(1\right) & \cdots & x_{n}{\left(1\right)}^{2}\\ 1 & x_{2}\left(2\right) & \cdots & x_{i}\left(2\right)x_{j}\left(2\right) & \cdots & x_{n}{\left(2\right)}^{2}\\ \vdots & \vdots & & \vdots & & \vdots \\ 1 & x_{1}\left(N\right) & \cdots & x_{i}\left(N\right)x_{j}\left(N\right) & \cdots & x_{n}{\left(N\right)}^{2} \end{array}\right], \end{array}$$

and this matrix (13) is evaluated only once, so the weight updates by L-M formula (5) can be evaluated with only varying error *e* that is recalculated at each epoch of training, so the the matrix multiplications and inversion with **J** are calculated only once for each retraining of QNU. However, the natural disadvantage of QNU is the exponentially increasing number of weights with number of inputs (e.g., QNU with *n* = 30 external inputs has *m* = 496 weights), so the inverse matrix operation in the L-M formula significantly slows down the retraining even if it is calculated once for all epochs. Also, choice of a proper technique for computation of precise inverse matrix may be an issue itself that can negatively influence the training technique. Therefore we may implement a weight-by-weight calculation approach (modified Levenberg-Marquardt adaptation) that avoids matrix inversion to calculate all neural weight updates by the L-M algorithm (as indicated in previous section and also used for MLP in that same subsection). The approach is shown in next subsection, where we show that Jacobian matrix (6) of static QNU can be calculated only once and that also the matrix inversion (5) can be avoided for QNU (Section 3.5). Thus the QNU becomes computationally fast enough for real-time calculation even on a PC (Ubuntu 12.04, Intel i5).

### 3.5. Quadratic Neural Unit with Modified Levenberg-Marquardt Adaptation

In this subsection, we present how we modified L-M algorithm to accelerate training of QNU by avoiding the inverse matrix computation. A general column of Jacobian matrix of QNU (10) that corresponds to a general weight *w*_*i*,*j*_ is *N* × 1 vector denoted as **j**_*i*,*j*_ and it is written as follows:

(14) $$\begin{array}{c} j_{i,j}=\left[\begin{array}{c} x_{i}\left(1\right)x_{j}\left(1\right)\\ x_{i}\left(2\right)x_{j}\left(2\right)\\ \vdots \\ x_{i}\left(N\right)x_{j}\left(N\right) \end{array}\right]. \end{array}$$

Then a single-weight increment is formally calculated according to original L-M formula (5) and because the term **j**_*i*,*j*_^*T*^ × **j**_*i*,*j*_ results in a scalar, we can use formula (9).

It is much faster to calculate individual vectors ${\tilde{j}}_{i,j}$ correspondingly to individual weights in a* for* loop using merely division (14) rather than to calculate all weight updates once by the original L-M formula with the inverse of a large matrix, that is, for QNU with too many inputs. Notice that all ${\tilde{j}}_{i,j}$ are calculated only once before the training in epochs starts and then we also calculate the weight updates only with varying **e** that is the only vector that is recalculated at every epoch in the modified L-M formula (14). As a result of the above modification of the L-M algorithm for QNU, the computation speed of QNU with retraining and prediction at every sample increased significantly (Figure 6). In other words, we are capable to implement the real-time prediction with retraining on a commonly available computational hardware without the need for more powerful one and the prototype of the software can be typically implemented either in Python or Matlab. The technique is further denoted as QNU predictor with MLM learning.

### 3.6. Quadratic Neural Unit with Normalised Gradient Descent Adaptation

In this subsection we present the normalized gradient descent algorithm [42, 43] for QNU with adaptation for prediction of lung tumor motion. This method of adaptation recalculates weights for every new sample. The weight update formula could be presented as follows:

(15) Δwk+1=μk·ek·∂yk∂wk=μk·ek·colxTk,

where *μ* is the normalised learning rate (16), *e* is prediction error (7), and the** colx** is vector of inputs, obtained from vector **x** (3) as shown in (12).

To improve stability of weight update system (15) during GD adaptation, the learning rate *μ* is normalized at every sample time as follows:

(16) $$\begin{array}{c} \mu \left(k\right)=\frac{\mu_{0}}{1+{\left(colx\left(k\right)\cdot {colx}^{T}\left(k\right)\right)}^{2}}, \end{array}$$

where *μ*_0_ stands for learning rate defined before the start of the simulation.

## 4. Experimental Analysis

### 4.1. Evaluation Criteria

Experimental analysis was performed on real respiration data of lung motion as described in Section 2 and using the two predictive models and the techniques described in Section 3. The objective of the analysis is to investigate the potentials for the prediction accuracy of 1 mm for prediction horizon of 1 s. We also present a more exhaustive study and comparison of static NN performance for prediction of lung motion using the real-time retraining technique. To evaluate the performance under the long-term condition required for clinical use, we highlight the results for prediction of the prediction horizons of 0.5 s and 1 s.

As the lung motion is measured in three axes, we analysed the predicting accuracy for various configurations by a 3D mean absolute error (MAE) as follows:

(17) $$\begin{array}{c} e_{3\text{D}}\left(k\right)=\sqrt{e_{1}{\left(k\right)}^{2}+e_{2}{\left(k\right)}^{2}+e_{3}{\left(k\right)}^{2}}, \end{array}$$

where *e*_1_, *e*_2_, and *e*_3_ are the predicting errors of corresponding axes, respectively. From the 3D error we can get the MAE with formula as follows:

(18) $$\begin{array}{c} \text{MAE}=\frac{1}{N}\overset{N}{\underset{k=1}{\sum }}e_{3\text{D}}\left(k\right), \end{array}$$

where *N* is the number of testing samples.

### 4.2. Experiments Setups

Also, the effect of various input configurations of the length of neural inputs *n* for the NN architectures (MLP in Figure 3, QNU in Figure 4) was studied. The optimum number of input-output training patterns *N*_train_ and number of neurons in the hidden layer *n*_1_ (in the case of MLP) were estimated after experiments. For MLP, we run each setup for the number of neurons in hidden layer as *n*_1_ = 1,2, 3,5, 7 (Figure 3) and this each instance of MLP predictor was repeated 10× from allays different random initial conditions. We highlight the results for prediction horizon *t*_pred_ = 1 s as summarized in Table 1.

### 4.3. Results

**Figure 5:**
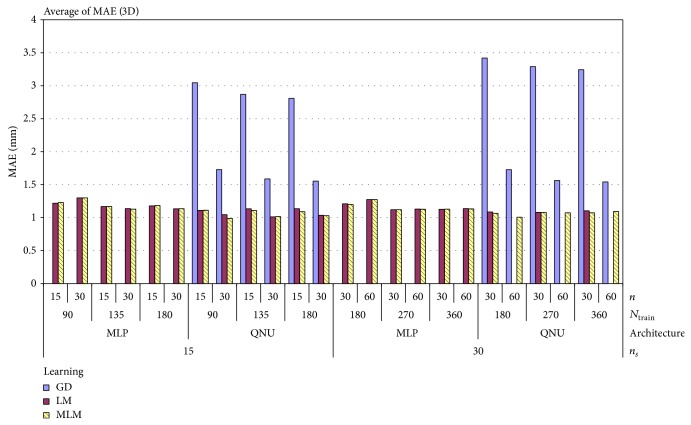
Mean absolute errors for 1-second prediction of 3D lung tumor motion with uncontrolled respiration for all predictors and simulation settings.

As it was specified in previous subsection, we ran 356 simulations for QNU and 2475 for MLP for real lung tumor motion time series shown in Figure 1. The results for all settings are shown in pivot chart of MAE in Figure 5. As we can see in that chart, results vary according to all parameters of simulation.

We concluded to setup 5 to 8 epochs for the sliding window retraining for MLP and 8 epochs for QNU as we could notice that the mean absolute error was not improved with more number of training epochs into the window especially for long-term prediction (up to 1 s). For pretraining before actual prediction we concluded to use 800 epochs for MLP and QNU using Levenberg-Marquardt adaptation and 400 epochs for QNU using gradient descent adaptation.

**Figure 6:**
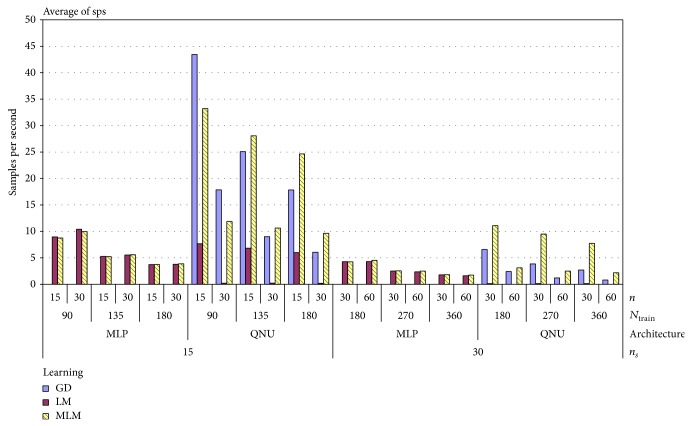
Computational speeds (sps) (PC, i5, Ubuntu) related to Figure 5 for all predictors and for all settings show the best suitability of QNU also for a possible real-time implementation.

We have to highlight that results of simulations with MLP depended more on random selection of initial weights than with QNU. The standard deviation of MAE of QNU was superior to MLPs as it is shown also in Table 2. This is most naturaly due to the known local minima issue of MLP with L-M algorithm while QNU is linear in its parameters, so QNU features a single minimum optimization problem. The lowest MAE was achieved by QNU with MLM adaptation and sampling 15 Hz as it is shown in Table 3. And in general, it is possible to say that simulations with smaller size of *N*_train_, that is, covering the range of about two respiration cycles, have better results. Difference of the MAE between using L-M and MLM can also be caused by initial random weights. However, the initial random weights were important to verify the general validity of this prediction approach. Also in general we can see from Figure 5 that QNU was more accurate than MLP. Figure 6 shows the pivot chart of computational speeds of all simulations. The higher the value on *y*-axis means the higher the computational speed. The fastest prediction was achieved with GD and MLM in combination with QNU for sampling 15 Hz, but the accuracy of GD with QNU was much worse than on with MLM. For almost of all used settings, the MLM was the fastest learning algorithm as it avoids inverse Jacobian matrix calculation. For the MLP predictor, the difference in computational speed of MLM and L-M was not that high as the Jacobian matrices of MLP were not so large here. The computation speed of QNU with MLM is significantly fastest as QNU calculates Jacobian only once and MLM avoids its inversion. The most accurate predictions and also the second fastest ones were obtained for QNU with the slower sampling 15 Hz, faster sampling 30 Hz, and with MLM learning algorithm as it is shown in Table 2. The prediction including retraining with QNU performed (PC, Ubuntu 12.04, Intel i5) in average 16 samples per second for one time series that is faster than real time. QNU performed also statistically better for all setups than MLP as regards the mean and standard deviation of MAE as it is shown in Table 3.

## 5. Discussion

As shown in Section 4.3, the results vary according to big amount of simulation settings. So far, we found the best algorithm for lung tumor motion prediction to be the QNU in combination with MLM. This prediction model achieved better accuracy than MLP and also it was fast in comparison with MLP model. Another advantage of QNU was better independence from initial weights for Levenberg-Marquardt algorithm. The choice of initial weights affected the prediction precision of MLP models (because of local minima issue mentioned earlier) and that accuracy issue can be a crucial problem for real-time usage of L-M algorithm with MLP predictors. According to our research that is presented in this paper, we can recommend the QNU predictor with MLM algorithm as a more suitable method than MLP or other reviewed approaches for fast respiratory time series prediction. However, the MLP predictors shall be further investigated as they are, in principle, capable of very high prediction accuracy and other suitable learning algorithm shall be investigated. For real implementations in a near-future, the computational speed (of MLP and of other approaches) might be significantly improved by nowadays spreading chipset on board and FPGA technologies. Our proposed prediction method is based on real-time retraining that can capture varying dynamics of a patient respiration. It shall be highlighted that our method was applied to lung tumor motion without any control of patient respiration. This implies that the dynamics of the patient respiration was varying unexpectedly. As regards instant variations of respiration dynamics and unexpected moves of a patient, we focus our research toward adaptive novelty detection for estimation of actual prediction accuracy [44]. Such approach seems to be promising for further improvement of prediction accuracy and for instant detection of unexpected moves with prospects to intensity modulated radiation tracking therapy.

## 6. Conclusions

In this paper, we have proposed and investigated real-time series predictive models of lung tumor 3D time series. A MLP with one hidden layer and quadratic neural unit were proposed and studied as predictive models. The studied learning rules for the models were the gradient descent adaptation (GD) and Levenberg-Marquardt batch optimization implemented as real-time retraining technique. We further modified L-M algorithm for faster real-time calculation. We demonstrated and compared the predictive capability of the models and algorithms on respiratory 3D lung tumor motion time series for real-time prediction. For the GD and L-M algorithm, we can conclude the superiority of QNU over MLP as regards the accuracy and real-time computational efficiency and reproducibility of the results. The in-parameter linearity of QNU avoided local minima issue during optimization while the initial weight setup of MLP importantly affects retraining accuracy for these comprehensible learning algorithms. The prediction results obtained by the predictive models satisfied the goals of our work for the prediction accuracy of 3D MAE of 1 mm for 1-second prediction horizon while the computational time was well shorter than the real treatment time.

## Figures and Tables

**Figure 7:**
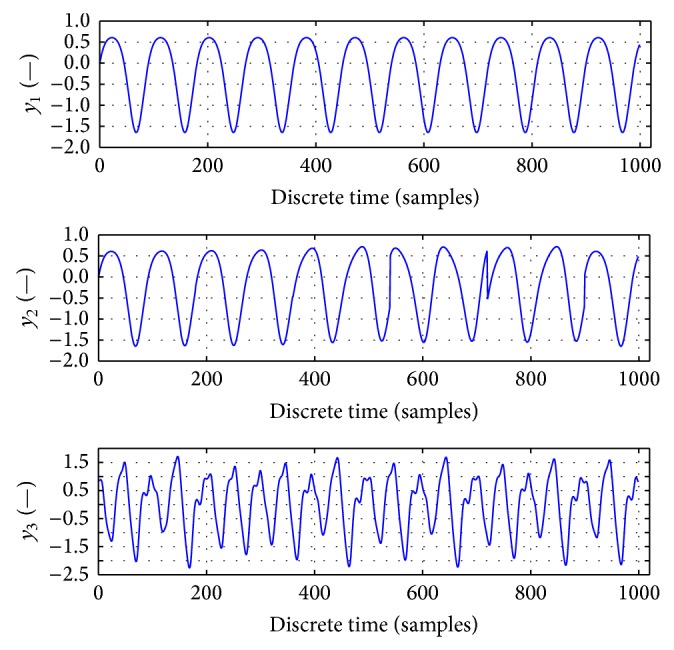
Artificial time series featuring nonlinear dynamics with random perturbations. Main frequency components correspond to respiration when considering sampling of 30 Hz.

**Figure 8:**
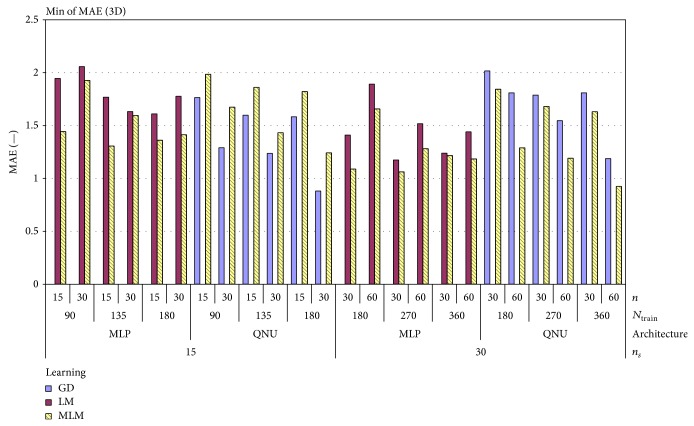
3D MAE for artificial data from Figure 7, total of 2826 simulation experiments.

**Figure 9:**
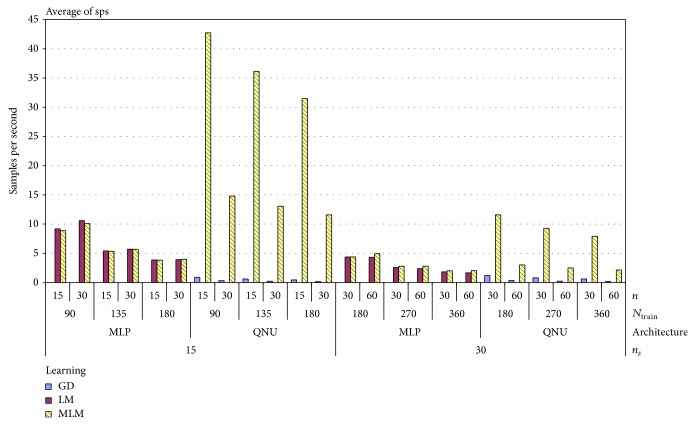
Computational speeds for MAE for artificial data computations shown in Figure 8.

**Figure 10:**
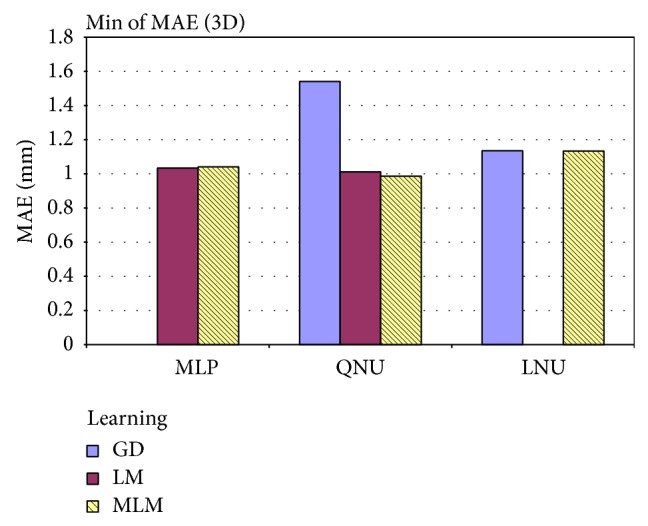
Minimum of MAE for 1-second prediction of lung tumor motion in 3D with uncontrolled respiration (out of all experiments for linear (LNU) and nonlinear predictors (QNU, MLP)).

**Figure 11:**
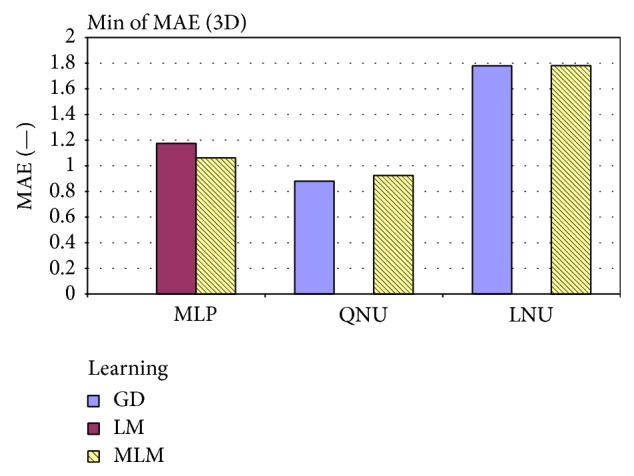
Minimum of 3D MAE for artificial data from all experiments for linear (LNU) and nonlinear predictors (QNU, MLP).

**Table 1:** General configuration for the predicting models (all for 1-second prediction horizon *t*, *n*…(3), *N*_train_…Figure 2).

Model	Learning algorithm	Sampling (also *n*_*s*_)	* n *[samples]		*N*_train_[samples]		
MLP	L-M	15	15	30	180	270	360
		30	30	60	90	135	180

QNU	L-M	15	15	30	180	270	360
		30	30	60	90	135	180

QNU	GD	15	15	30	180	270	360
		30	30	60	90	135	180

**Table 2:** The best achieved results of MAE [mm] for 3D lung tumor motion prediction with uncontrolled respiration and the setups for QNU and MLP architectures (MLPs (Figure 3) were investigated for *n*_1_ = 1,2, 3,5, 7.

Architecture	Learning	*N*_train_	*n*	*n*_*s*_	µ	Epochs 0	Epochs	MAE [mm]	*σ*(MAE) [mm]	sps	Count of trials
QNU	MLM	90	30	15	5.00*E* − 05	800	8	**0.987**	**0.001**	**16.05**	42

MLP	LM	180	30	15	0.01	800	8	1.034	0.033	3.11	150
	MLM	270	60	30	0.01	800	8	1.041	0.039	1.76	90

**Table 3:** The statistical comparison of MAE [mm] for MLP versus QNU over all various setups (on the 3D lung tumor motion prediction for prediction horizon of 1 second).

Learning	Data	Architecture		
		MLP	QNU	LNU
GD	Min of MAE		1.54	
	Average of MAE		2.33	
	Standard deviation of MAE		0.75	

LM	Min of MAE	1.03	1.01	
	Average of MAE	1.18	1.08	
	Standard deviation of MAE	0.08	0.04	

MLM	Min of MAE	1.04	**0.99**	1.13
	Average of MAE	1.18	**1.06**	1.28
	Standard deviation of MAE	0.08	**0.04**	0.13

## Appendix

### Artificial Data Experiment

Up to date, we unfortunately have not obtained another real lung tumor 3D motion data with uncontrolled patient respiration. Thus we generated three artificial time series shown in Figure 7 to validate the proposed approach. We created first time series *y*1 by a nonlinear function (A.1) as follows

(A.1) yt=5·sin2π3t−sin2π/31/2,t=k30, k=1,…,1000,

and the second time series *y*_2_ was generated by (A.1) with randomly varying main frequency every 3 samples. The third time series *y*_3_ was generated using the famous chaotic Mackey-Glass equation as follows:

(A.2) $$\begin{array}{c} \frac{dy\left(t\right)}{dt}=\frac{b\cdot y\left(t-\tau \right)}{1+y{\left(t-\tau \right)}^{10}}-g\cdot \left(t\right), \end{array}$$

where *t* denotes continuous time, and the chaotic behavior was generated by the setup of *b* = 0.2, *g* = 0.1, and the lag *τ* = 17.

The results on 3D MAE and speed of computation are shown in Figures 8, 9, 10, and 11. These results confirm our achievements on real data sets, in particularly, the QNU appears accurate and efficient predictor in comparison to conventional MLP networks when GD, L-M, or MLM learning are used.

For completeness of this study, we show results achieved with (with real-time retraining) linear predictor LNU (linear neural unit) that demonstrates the need for nonlinear predictive models because of prediction accuracy.

For validation of result reproducibility, we also performed the computations on artifical data with another HW (PC, Windows 7, i7), so the computational speeds can differ from the ones achieved for real data. The results on artifical data confirm our achievements with real tumor motion data with uncontrolled respiration.
